# Why people were less compliant with public health regulations during the second wave of the Covid-19 outbreak: The role of trust in governmental organizations, future anxiety, fatigue, and Covid-19 risk perception

**DOI:** 10.1007/s12144-021-02059-x

**Published:** 2021-07-13

**Authors:** Cristiano Scandurra, Vincenzo Bochicchio, Pasquale Dolce, Paolo Valerio, Benedetta Muzii, Nelson Mauro Maldonato

**Affiliations:** 1 Department of Neuroscience, Reproductive Sciences and Dentistry, University of Naples Federico II, Via Sergio Pansini 5, 80131 Naples, Italy; 2 Department of Humanistic Studies, University of Calabria, Via Ponte Bucci Cubo 18/C, 87036 Rende, Italy; 3 Department of Public Health, University of Naples Federico II, Via Sergio Pansini 5, 80131 Naples, Italy; 4 Department of Humanistic Studies, University of Naples Federico II, Via Porta di Massa 1, 80133 Naples, Italy

**Keywords:** Political trust, Fatigue, Future anxiety, Protective behaviors, Public health, Covid-19

## Abstract

Trust in governmental organizations is a crucial factor in terms of encouraging people to conform to public health regulations, such as those recommended to slow down the spread of SARS-CoV-2. However, trust in governmental organizations tends to decline over time, reducing the compliance with public health regulations. This study aimed at exploring, first, the role of future anxiety and fatigue as serial mediators of the relationship between trust in governmental organizations and protective behaviors, and, secondly, the role of Covid-19 risk perception as a moderator between fatigue and protective behaviors. A total of 948 Italian participants (302 males and 646 females), ranged from 18 to 80 years (*M* = 27.20, *SD* = 11.01), answered an online survey during the second wave of the Covid-19 outbreak. A moderated serial mediation model was performed using a structural equation modeling. The results indicate that: (1) a higher trust in Italian governmental organizations was associated with a greater compliance in terms of adopting protective behaviors; (2) a lower trust in Italian governmental organizations increased anxiety about the future which, in turn, raised levels of fatigue, leading, finally, to a reduction in the levels of protective behaviors; and (3) as the perceived risk related to Covid-19 increased, the effect of fatigue on protective behaviors decreased. The findings of the current study may provide indications for public health policy on how to increase compliance with the recommended behaviors to be adopted in order to decrease the spread of the SARS-CoV-2.

## Introduction

The coronavirus disease 2019 (Covid-19), an infectious respiratory illness caused by the severe acute respiratory syndrome coronavirus 2 (SARS-CoV-2), broke out in December 2019 in Wuhan, Hubei province, in China. Within a few months, it spreads globally and very rapidly, becoming a public health emergency of international concern (Phelan et al., 2020). Indeed, in March 2020, the World Health Organization recognized Covid-19 as a pandemic. Although at the moment of the current study some vaccines had been authorized and specific social categories (e.g., healthcare workers) had started to be vaccinated, vaccines were not yet widely available. In the absence of an immediate large-scale vaccine, non-pharmaceutical interventions were recommended worldwide as the only public health regulations which could be effective in slowing down the spread of the virus, in particular social distancing, hand washing and wearing masks in public places (Jovančević & Milićević, 2020; Makhanova & Shepherd, 2020; Zhou et al., 2020). However, successful implementation of these measures depended on compliance with the regulations and support from the public (Anderson et al., 2020).

A factor that is crucial in encouraging people to follow the public health regulations is trust in governmental organizations, which can be defined as the confidence of individuals in the government and their satisfaction with the government performance (Bouckaert & Van de Walle, 2003). More specifically, trust in governmental organizations represents a valid indicator of social capital (Kwon et al., 2013). Indeed, during times of uncertainty, like those experienced during the Covid-19 pandemic, trust in governmental organizations is vital for the social contract between individuals and government (Toya & Skidmore, 2014), as it was demonstrated that governments with higher levels of trust from the public can govern more effectively than their counterparts (Cooper et al., 2008). To this end, people seem to have more confidence in their governments when they perceive that the government has the ability, expertise, and technical knowledge to make the best decisions for the public interest (Gozgor, 2021). Furthermore, it was also reported that effective public communication from the social institutions, as well as clear and unambiguous behavioral indications, may reduce the maladjusted behaviors by buffering the intolerance for uncertainty (Bochicchio et al., 2021). Thus, in terms of related psychological constructs, trust in government may elicit a spontaneous sociability and, as a consequence, cooperative and altruistic behaviors in social activities (Uslaner, 2018; Zmerli & Van der Meer, 2017). Additionally, in line with the source model of group threat (Greenaway & Cruwys, 2019), it seems that external threats increase societal trust due to the shared need to overcome the disaster by working together (Toya & Skidmore, 2014), as groups experiencing external threats tend to respond by tightening ingroup ties and increasing active participation.

In line with the above references, previous research has reported evidence of the role of trust in governmental organizations in terms of following recommended behaviors to avoid the spread of swine flu (Rubin et al., 2009) and Ebola (Blair et al., 2017), as well as in getting vaccinated against seasonal influenza (Verger et al., 2018). Recent evidence has also suggested that trust in governmental organizations represents a crucial factor even in terms of compliance with the public health regulations suggested to slow down the spread of SARS-CoV-2 (e.g., Olagoke et al., 2020; Sibley et al., 2020; Storopoli et al., 2020). However, research conducted during previous epidemics (e.g., swine flu) demonstrated that, while trust in governmental organizations is very high during the initial stages of the pandemic, it tends to decline over time, decreasing the level of compliance with public health regulations (Bangerter et al., 2012; Quinn et al., 2013). This seems to be the case of Italy, which is the context of the current study, where the citizens have protested, in some cases violently, as soon as the central government has rolled out new protective measures (e.g., lockdowns, the closure of educational institutions and commercial activities, and the prohibition of public events and travel) to prevent the further spread of the virus during the so-called “second wave” of the Covid-19 outbreak, which broke out in October 2020 (Lowen, 2020).The second wave has been attributed to the fact that, after the first lockdown had occurred in March 2020, measures were lifted across the EU during the summer (Bontempi, 2020). Indeed, since the beginning of September 2020 Italy has started to register more than 2000 new cases per day (Buonsenso et al., 2020).

Notwithstanding these data, potential mediators able to elucidate the possible reasons why a low trust in governmental organizations should lead to a decrease in the likelihood of adopting recommended protective behaviors are still unclear. In the following paragraphs, we will propose the role of fatigue and future anxiety as two potential mediators.

As regards fatigue, the World Health Organization (WHO, 2020) published a report immediately after the beginning of the second wave as a response to EU Member States who were reporting that populations seemed to be highly fatigued and less compliant with the recommended protective measures. The WHO (2020) introduced the concept of “pandemic fatigue,” defined in line with previous studies (Masten & Motti-Stefanidi, 2020; Morrison et al., 2018) as a natural long-term response to the adversity caused by a pandemic whose main outcome is a demotivation in terms of engaging in recommended protective behaviors. “Pandemic fatigue” could also be interpreted as a sort of “societal burnout” (Queen & Harding, 2020), i.e. a condition of emotional, physical, and mental exhaustion caused by excessive and prolonged stress due to the pandemic restrictions. Indeed, it has been widely demonstrated that restrictions have produced unprecedented stressors (e.g., threats to personal safety, feelings of being out of control, loneliness, exhaustion) which have negatively affected emotional, physical, and mental wellbeing of people (Ammar et al., 2020; Mattioli et al., 2020; Restauri & Sheridan, 2020). Recently, taking into account the psychological impact of Covid-19 pandemic and in line with the concept of “societal burnout,” Teixeira da Silva (2021) has coined the term “Coronex” to refer to the long-term exhaustion that is caused by an excessive fatigue accumulated over time. According to this concept, the fatigue resulting from the Covid-19 pandemic has weakened people, depriving them of energy and motivation and generating an entrenched sense of hopelessness and despair (Teixeira da Silva, 2021). Thus, the concept of “societal burnout” has a noteworthy heuristic value, because it allows an understanding of the different behaviors observed in the population during the second wave of the Covid-19 outbreak as a unitary response to the health crisis, i.e. practicing poor self-care and avoiding self-protecting behaviors, feeling exhausted and demotivated, and experiencing deep and prolonged anxiety and worry. It is also interesting to highlight that several studies on job burnout have shown that “organizational trust,” that is the perception of the support provided by an organization and the belief that the organization is honest, transparent, and reliable (Shockley-Zalabak et al., 2000), is associated with worker burnout (Özgür & Tektaş, 2018), since the lower is the trust in the organization and its managers the higher is the risk of burnout.

In addition to fatigue, it is plausible to suppose that the trust in governmental organizations is associated with other emotional and behavioral variables that we can group together heuristically within the construct of “societal burnout,” such as anxiety about the future. Indeed, the uncertainty caused by the Covid-19 outbreak has had a profound negative impact on people’s mental well-being, producing stress, anxiety, depression, and suicidal ideation (Maldonato et al., 2020; Garfin et al., 2020; Killgore et al., 2020; Lima4 et al., 2020). Since there are no certainties in relation to the end of the pandemic, feelings of uncontrollability may dramatically increase and this, in turn, may cause anxiety as a common response to a stressful situation (Giallonardo et al., 2020; Liu et al., 2020; Usher et al., 2020). Additionally, the Covid-19 outbreak has exacerbated economic and social problems, such as unemployment and economic collapse, and this has generated anticipatory fears which have, in turn, increased anxiety about the future (Gasparro et al., 2020; Paredes et al., 2021).

Previous research has demonstrated that uncertainty and worry prove to be associated with fatigue, suggesting that greater levels of uncertainty increase levels of fatigue (Nitschke et al., 2020). Additionally, previous studies have also reported that trust in governmental organizations may represent a crucial factor in determining people’s attitudes and behaviours (Hocevar et al., 2017), as it may reduce anxiety and promote compliance with public health regulations (Hornsey & Esposo, 2009), linking trust in governments with future anxiety. Indeed, people with low levels of trust in governmental organizations may perceive that the situation is unpredictable and out of control, thereby increasing feelings of anxiety about the future.

Moreover, beyond mediators, it is hypothesized that there might be potential moderators which may act to increase or decrease levels of compliance with public health regulations. To investigate this possibility, the WHO (2020) stressed the proposition that, although epidemiological data highlight the strong risk and negative health and social consequences of being infected by SARS-CoV-2, the perceived threat of the virus may decrease over time due to the habit to its existence. In this respect, previous research has demonstrated that the risk perception related to a pandemic represents a protective factor contributing to an increase in compliance with the recommended preventive measures (Cowling et al., 2010; Prete et al., 2020; van der Weerd et al., 2011). This idea is in line with the Protection Motivation Theory (PMT), which suggests that the public perception of the severity of a health threat, and the individual’s vulnerability to it, determines the risk perception in relation to a specific disease, and this leads people to engage in either healthy or unhealthy behaviors (Rogers, 1975).

Thus, since the second wave of the Covid-19 outbreak seems to have produced a decrease in the levels of compliance with the public health regulations prescribed to slow down the spread of the virus, this study aimed at testing a moderated serial mediation model where future anxiety and fatigue have been considered as two potential serial mediators of the relationship between trust in governmental organizations and protective behaviors, and Covid-19 risk perception as a moderator between fatigue and protective behaviors.

Specifically, based on previous works that have demonstrated the direct relationship between trust in governmental organizations and compliance with public health regulations (Blair et al., 2017; Olagoke et al., 2020; Rubin et al., 2009; Sibley et al., 2020; Storopoli et al., 2020; Verger et al., 2018), we hypothesized that a greater trust in governmental organizations would increase the levels of protective behaviors (Hypothesis 1). Next, based on previous studies which have identified associations between trust in governmental organizations and future anxiety (Hornsey & Esposo, 2009), uncertainty and fatigue (Nitschke et al., 2020), and fatigue and low compliance with public health regulations (WHO, 2020), we hypothesized that the relationship between trust in government organizations and protective behaviors would be mediated by both future anxiety and fatigue (Hypothesis 2). In particular, we expected that lower levels of trust in governmental organizations would increase anxiety about the future which, in turn, would increase the levels of fatigue; then, that higher levels of fatigue would lead to a reduction in the levels of protective behaviors. Nonetheless, based on PMT (Rogers, 1975) and on most recent evidence about the role of risk perception as a protective factor during a pandemic (Cowling et al., 2010; Prete et al., 2020; van der Weerd et al., 2011), we also hypothesized that the perception of Covid-19 risk would buffer the negative effects that fatigue may have on protective behaviors, increasing the levels of the protective behaviors adopted (Hypothesis 3).

In addition, several socio-demographic factors were considered in the current study, as some of these factors have been proved to increase or decrease the likelihood of adopting protective behaviors during pandemics (Bish & Michie, 2010). Specifically, older individuals (e.g., Jones & Salathé, 2009; Lau et al. 2007), women (e.g., Brug et al., 2004; Quah & Hin-Peng, 2004), people with a higher level of education and socio-economic status (SES; Lau et al., 2007), and those who have come into direct contact with the virus (i.e., personal knowledge of people who had been infected by or had died due to Covid-19 or those who have been infected with the SARS-CoV-2; Bochicchio et al., 2021) are generally more likely to adopt precautionary behaviors than their counterparts.

## Methods

### Procedures

The current study used a cross-sectional online survey administered via the Qualtrics survey software. The survey was launched on social media (e.g., Facebook) between 25th October and 15th November 2020, which was the peak period of the second wave of the pandemic in Italy. The participants were recruited through a snowball sampling recruitment procedure, in that we encouraged them to share the survey with their contacts. In this dissemination phase, we made every effort to cover all the Italian regions by sharing the survey with different regional groups constituted by a large number of members.

By clicking on the link provided, the participants were directed to the first page of the survey where they could read the informed consent form, the objectives, benefits and risks of the study, and information about the researchers. The participants were informed about the anonymity of the survey, the right to withdraw from it, and the time needed to complete it (approximately 20 min). To avoid missing data, all questions had to be completed in order to proceed through the survey. At the end of the survey, the participants were also informed about the opportunity of receiving a short report on the study once the data had been analyzed and were asked to send their personal e-mail addresses to the principal investigator accordingly.

The study was approved by the ethical committee of the University of Calabria, as well as designed in accordance with the Declaration of Helsinki on the Ethical Principles for Medical Research Involving Human Subjects and in accordance with the EU General Data Protection Regulation.

### Participants

The inclusion criteria for participation in the study were: (1) being at least 18 years old, the Italian age of consent; (2) living in Italy; and (3) not being a health professional. As regards this last inclusion criterion, we decided not to include health professionals as we assumed that, although health professionals may have similar experiences compared with the general population, they are expected to engage in protective behaviors to a greater extent than the general population, regardless of the predictors considered in the current study.

A total of 1001 participants took part in the survey. Among these, 43 did not satisfy one or more of the inclusion criteria. Furthermore, 10 participants presented standardized scores higher than 3.29 or lower than −3.29 on at least one measure, and therefore they were considered as outliers following the recommendations by Tabachnick and Fidell (2001). Thus, the final sample was composed of 948 Italian participants (302 males and 646 females). The participants ranged in age from 18 to 80 years old (*M* = 27.20, *SD* = 11.01). Overall, 33.2% (*n* = 315) were highly educated (≥ a college degree) and 72.7% (*n* = 689) declared that they had a medium SES. Finally, 82.4% (*n* = 781) personally knew an infected person, 21% (*n* = 199) personally knew a person who had died due to Covid-19, and 4.5% (*n* = 43) had been diagnosed with Covid-19.

### Measures

#### Socio-Demographic Information

The socio-demographic characteristics assessed in the current study included gender identity (women, men, and other), age, level of education (1 = high school or lower; 2 = college or higher), SES (from extremely low to extremely high on a 5 point-Likert scale), personal knowledge of someone who had been infected by or had died due to Covid-19 (yes vs. no), and a personal diagnosis of Covid-19 (yes vs. no).

#### Trust in Governmental Organizations

Trust in governmental organizations was measured with the Citizen Trust in Government Organizations scale (CTGO; Grimmelikhuijsen & Knies, 2017), a 9-item questionnaire assessing through three subscales the extent to which individuals perceive government organizations as capable and effective (i.e., Perceived Competence), motivated to act in the public interest (i.e., Perceived Benevolence), and sincere (i.e., Perceived Integrity). Grimmelikhuijsen and Knies (2017) proposed that the focal entity (e.g., a particular municipality) and the specific public task (e.g., air quality policy) may be varied and then added to the list of items. In the current study, the items had been formulated to focus on the role of Italian political/administrative institutions in relation to the management of the second wave of the Covid-19 outbreak. An example item is “As regards the management of the second wave of the Covid-19 outbreak, Italian political/administrative institutions are capable?” The response options ranged from 1 (strongly disagree) to 5 (strongly agree), with higher scores indicating a higher trust in governmental organizations. The alpha coefficients for the current sample were .85, .84, and .88 for the three subscales, respectively.

#### Anxiety about the Future

The Dark Future Scale (DFS; Zaleski et al., 2019) was used to assess the tendency to think about the future with uncertainty and anxiety. The DFS is a 5-item questionnaire and the response options ranged from 0 (decidedly false) to 6 (decidedly true), with higher scores indicating a greater anxiety about the future. An example item is “I am afraid that in the future my life will change for the worse.” The alpha coefficient for the current sample was 0.88.

#### Fatigue

Pandemic-related fatigue was measured with the Fatigue Assessment Scale (FAS; Michielsen et al., 2003), a 10-item questionnaire evaluating the physical and mental symptoms of chronic fatigue. With the aim of aligning the scale to the objectives of the current study, the participants were asked to think about the second wave of the Covid-19 outbreak in answering the questions. Specifically, the instructions were: “The following statements refer to how you feel about the current situation due to Covid-19. In answering, therefore, think of the current second wave of the infection.” The response options ranged from 1 (never) to 5 (always), with higher scores indicating greater fatigue. An example item is “I am bothered by fatigue.” The alpha coefficient for the current sample was 0.90.

#### Covid-19 Risk Perception

The Covid-19 Perceived Risk Scale (CPRS; Yıldırım & Güler, 2020) was used to assess the cognitive (i.e., probability and severity of outcomes evaluated from extant information) and emotional (i.e., worry, concern, and fear) aspects of perceived personal risk related to Covid-19. The CPRS is an 8-item questionnaire whose response options range from 1 (negligible) to 5 (very high), with higher scores reflecting higher levels of personal risk related to Covid-19. Example items are “What is the likelihood that you would acquire the Covid-19?” or “How worried are you about contracting Covid-19?” The alpha coefficient for the current sample was 0.71.

#### Protective Behaviors in Relation to Covid-19

To assess the protective behaviors prescribed by governments in relation to Covid-19 we used the Routine Protective Behaviors (RPB) subscales of the Protective Behaviors in relation to Covid-19 Scale (Riad et al., 2020). The RPB measures the extent to which people adopt five protective behaviors, namely: keeping their hands clean, not participating in parties, avoiding travelling unless necessary, avoiding visiting parents or friends unless necessary, and covering their mouth and nose when in public. The response options range from 1 (not at all like me) to 5 (just like me), so that higher scores reflect a greater level of adoption of protective behaviors. The alpha coefficient for the current sample was 0.75.

### Statistical Analyses

All the statistical analyses were performed using the R software environment for statistical computing. The level of significance for all the statistical tests was set at α = .05.

The bivariate correlations between the main variables of the study (trust in governmental organizations, anxiety about the future, fatigue, Covid-19 risk perception and protective behaviors) were calculated through the Pearson coefficient.

Serial mediation analysis was conducted to test the first and second hypotheses of the current study, while moderation analysis was conducted to test the third hypothesis. We used the *lavaan R package* for the structural equation modeling (Rosseel, 2012) to fit the moderated serial mediation model. Bollen-Stine bootstrapping was used for the statistical tests, and therefore bootstrap standard errors were computed using model-based bootstrapping. The number of bootstrap samples was set at 5000. We specified in the model all the subscales of the CTGO as measures of a common latent factor (i.e., trust in governmental organizations). To evaluate the mediation and the moderated-mediated effect, we tested for the statistical significance of the coefficient of the Mediation Effect (ME) and the Index of Moderated Mediation (IMM).

As socio-demographic variables may influence the adoption of protective behaviors (e.g., Bish & Michie, 2010), we adjusted the models by including different potential confounding variables, namely age, gender identity, level of education, SES, personal knowledge of people who had been infected by or had died due to Covid-19 and a personal diagnosis of Covid-19.

## Results

### Descriptive Statistics and Bivariate Correlations

The means, standard deviations, reliabilities for the variables, and bivariate correlations are shown in Table 1. The results highlight that all the dimensions of trust in Italian political/administrative institutions were correlated negatively with future anxiety and positively with protective behaviors. Perceived competence and integrity, but not benevolence, were negatively correlated with fatigue. Future anxiety was positively correlated with fatigue, Covid-19 perceived risk, and protective behaviors. Fatigue was positively correlated with Covid-19 perceived risk but not with protective behaviors. Finally, Covid-19 perceived risk was positively correlated with protective behaviors.

**Table 1:** Descriptive statistics and correlations among trust in governmental organizations, future anxiety, fatigue, Covid-19 risk perception, and routine protective behaviors

Scales	1	2	3	4	5	6	7	Mean	SD	Skewness	Kurtosis
1. CTGOS Benevolence	–							7.24	2.54	0.12	−0.49
2. CTGOS Competence	0.68***	–						7.82	2.79	0.04	−0.29
3. CTGOS Integrity	0.63***	0.73***	–					6.66	2.69	0.42	−0.67
4. Future Anxiety	−0.09**	−0.09**	−0.13***	–				3.43	1.47	−0.25	−0.67
5. Fatigue	−0.03	−0.11**	−0.09**	0.51***	–			25.77	8.84	0.45	−0.71
6. CPRS	0.05	−0.02	−0.01	0.25***	0.22***	–		25.17	4.55	−0.04	0.14
7. RPB	0.07*	0.11***	0.07*	0.07*	0.02	0.23***	–	20.47	3.29	−0.91	0.95

*p < 0.05; **p < 0.01; ***p < 0.001

*CTGOS* Citizen Trust in Government Organizations Scale, *CPRS* Covid-19 Perceived Risk Scale, *RPB* Routine Protective Behaviors

### Direct and Indirect Associations between Trust in Governmental Organizations, Future Anxiety, Fatigue, and Protective Behaviors

**Fig. 1:**
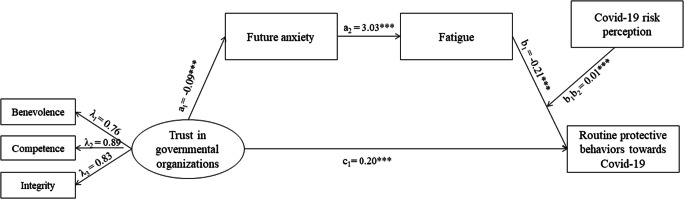
Results from the structural equation modeling of the hypothesized moderated serial mediation model. Standardized path coefficients are reported. *Notes*. Covariates were not reported for simplification reasons. ***p < 0.001

As shown in Fig. 1, and with respect to Hypothesis 1, the results indicate that trust in Italian political/administrative institutions was positively associated with the adoption of protective behaviors, *c* = 0.20, *p* < 0.001, *95% CI* [0.09, 0.31], confirming our hypothesis.

With regard to Hypothesis 2, we found that lower levels of trust in Italian political/administrative institutions increased anxiety about the future, *a*_*1*_ = −0.09, *p* < 0.001, *95% CI* [−0.15, −0.04] which, in turn, increased levels of fatigue, *a*_*2*_ = 3.03, *p* < 0.001, *95% CI* [2.71, 3.36]; furthermore, greater fatigue led to a reduction in the levels of adoption of protective behaviors, *b*_*1*_ = −0.21, *p* < 0.001, *95% CI* [−0.23, −0.19]. These results confirm our hypothesis.

The serial mediation effect of future anxiety and fatigue was statistically significant (*ME* = 0.06; *p* = 0.001).

#### Control Variables

Among the socio-demographic characteristics used as control variables, only being female (*b* = 0.59, *p* = 0.008), having a higher educational level (*b* = 1.15, *p* < 0.001), and a higher SES (*b* = 0.37, *p* = 0.040) proved to be associated with greater levels of adoption of protective behaviors.

### The Moderating Role of Covid-19 Risk Perception

**Fig. 2:**
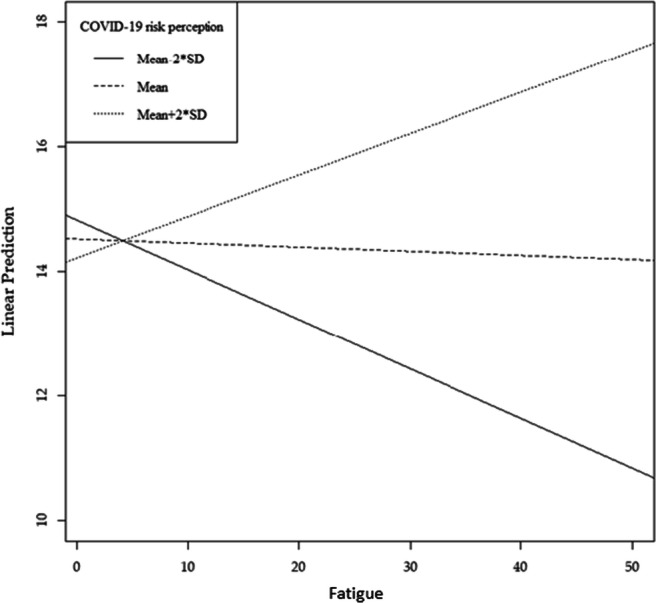
Conditional indirect effect of fatigue on protective behaviors as a function of Covid-19 risk perception

Concerning hypothesis 3, we found a significant and positive interaction between fatigue and Covid-19 risk perception on protective behaviors, *b*_*1*_*b*_*2*_ = 0.01, *p* < 0.001, *95% CI* [0.01, 0.02]. Indeed, as the perceived risk related to Covid-19 increased, the effect of fatigue on protective behaviors decreased (see Fig. 2), confirming our hypothesis. The moderation effect of fatigue on the adoption of protective behaviors was statistically significant (*IMM* = −0.01; *p* < 0.001).

## Discussion

The current study was aimed at assessing the role of the trust of the Italian population in governmental organizations, future anxiety, fatigue, and Covid-19 risk perception in relation to the compliance with public health regulations during the second wave of the Covid-19 outbreak. Our results suggest that a higher level of trust in Italian political/administrative institutions increased the level of compliance with public health regulations, while anxiety about the future and fatigue explain why a lower level of trust in governmental organization decreased the adoption of protective behaviors. Furthermore, a higher perceived risk in relation to Covid-19 decreased the negative effect of fatigue on compliance with public health regulations. To the best of our knowledge, this is the only Italian study assessing these relationships during the second wave of the Covid-19 outbreak. This was a challenging phase of the pandemic as individuals were fatigued and compliance with public health regulations seemed to decrease leading to an increase of infection rates, although the country was less affected in comparison with other EU countries (Bontempi, 2020).

In support of our first hypothesis, this study confirmed previous research concerning the association between trust in governmental organizations and compliance with public health regulations during the Covid-19 outbreak (Olagoke et al., 2020; Sibley et al., 2020; Storopoli et al., 2020). Indeed, a greater level of trust in political/administrative institutions seems to be one of the strongest predictors of the population’s level of compliance with public health regulations during a pandemic (Blair et al., 2017; Rubin et al., 2009; Verger et al., 2018). Conversely, a lower level of trust in governmental organization could make the control of the spread of the virus more difficult, as it might lead to a rejection of the official information and, as a consequence, a high degree of non-compliance with public health recommendations (Han et al., 2020).

However, the innovative finding of the current study mainly concerns the second hypothesis, or rather the serial mediating role of future anxiety and fatigue. Indeed, our results suggest that, when individuals have a low trust in governmental organizations, they tend to report high levels of anxiety about the future and, in turn, such anxiety increases the levels of fatigue. Such greater fatigue might explain the low level of compliance with public health regulations. This result seems to confirm the hypothesis that a lack of trust in governmental institutions may lead individuals to develop a high degree of uncertainty about the future, perceiving the social situation as unpredictable and out of control and, thus, developing a sort of “societal burnout” (Queen & Harding, 2020). Therefore, as suggested by Nitschke et al. (2020), uncertainty and related worries increase the perceived levels of fatigue which, in turn, as suggested by the WHO (2020), demotivates individuals in terms of their compliance with public health regulations. This means that governments should find effective ways to lead the population to perceive them as trustworthy, since high levels of trust would probably reinforce the individuals’ perceptions of having control over the situation, decreasing the perceived fatigue, and increasing the levels of compliance with recommended protective behaviors.

Notwithstanding this, in support of the third hypothesis of the current study, if citizens who do not trust governmental organizations maintain a high perception of risk, in this case related to Covid-19, the detrimental effect of fatigue on the adoption of protective behaviors decreases, so reducing the risk of any non-compliance with public health regulations. This finding confirms previous studies which have highlighted that perceiving a risk related to a pandemic may be a protective factor contributing to an increase in compliance with public health regulations (e.g., Cowling et al., 2010; Prete et al., 2020; van der Weerd et al., 2011). However, this does not mean that we should raise the levels of risk perception related to Covid-19 to increase the likelihood that people will become more compliant with public health regulations. Indeed, previous research has demonstrated that people with high levels of health anxiety and risk perception tend to demonstrate non-rational behaviors, such as avoiding attendance at a clinic, even in case of necessity, because clinics are perceived as a source of contagion (Lee, 2014; Taylor, 2019). Therefore, as suggested below, in accordance with Sobkow et al. (2020), we believe that governmental actions should be addressed to lower anxiety levels but, at the same time, governments should clearly report the real risks related to Covid-19.

Interestingly, our results also suggest that being female, having a higher educational level and a higher SES were the only socio-demographic characteristics that proved to be associated with a greater compliance with public health regulations. This means that, if we consider the negative aspects of the relationships investigated (i.e., the low levels of trust in governmental organization, and the high levels of future anxiety and fatigue), potential public health interventions aimed at increasing the possibility that most individuals will be compliant with public health regulations should be particularly addressed at men and those with low educational levels and a low SES. These results are in line with previous studies which have highlighted that both gender and educational and social status influence the degree of adoption of protective behaviors, with men and individuals with lower levels of literacy being less likely to be compliant (Carlucci et al., 2020; Haque et al., 2020; Lüdecke & von dem Knesebeck, 2020).

### Limitations

Our findings should be read in light of important limitations. First, the cross-sectional nature of the study has prevented us from making definitive conclusions about the temporality and causality of the relationships explored between variables. Future studies should implement longitudinal designs to determine the cause-effect relationships of trust in governmental organizations, future anxiety, fatigue, and Covid-19 risk perception with protective behaviors. Secondly, despite the large size of the sample, it is not representative of the whole Italian population, which does not allow any generalization of our findings to the Italian context. Additionally, although statistical models were adjusted for several socio-demographic factors, the sample was unbalanced in terms of gender, with a preponderance of women. This may have influenced the results, as women are generally more likely than men to adopt precautionary behaviors during pandemics (Bish & Michie, 2010). Future studies should try to recruit more gender-balanced samples. Furthermore, as the participants were recruited only in Italy, our results should be understood as culturally characterized. Future studies should consider replicating this study in other contexts, analyzing any potential cultural differences that we were unable to capture. Thirdly, although scientific reports have registered a dramatic decrease in compliance with public health regulations during the second wave of the infection (e.g., WHO, 2020), we have not been able to compare the participants’ level of compliance with such regulations with that achieved during the first wave of the infection. However, the aim of the current project was not to explore any differences between the two waves, but rather to analyze the potential mechanisms that might lead people to become less compliant.

### Implications for Public Health Policy

Despite these limitations, our study may have some important implications for public health policy. Indeed, according to our findings, which have highlighted that a low compliance with public health regulations may depend on the interaction of several factors, we can provide some suggestions on how to increase levels of compliance with the behaviors recommended to be adopted to decrease the spread of SARS-CoV-2.

In this respect, we believe that the most direct implication of our research concerns the communication strategies and behaviors that governmental representatives should adopt to increase levels of awareness of the need to adopt routine protective behaviors both for individuals themselves and society in general. To be effective in this action, in accordance with our results, governmental organizations should communicate and behave in ways that encourage the public to perceive them as trustworthy. Previous research has demonstrated that governments are more likely to be perceived as trustworthy and to inspire trust by communicating with the public in a clear and sensitive manner, providing an impression of competence and legitimacy, and clearly reporting information on the social, economic, and health impacts of the pandemic (Lalot et al., 2020). Furthermore, previous studies have highlighted that compliance with governmental regulations is higher when the political leaders construct a shared social identity and are perceived as acting in the general interest (Haslam & Reicher, 2017; Van Bavel et al., 2020).

Therefore, we believe that the leaders of the Italian governmental organizations should adopt a coordinated approach, reaching out beyond their political alliances and acting in the collective interest. Accordingly, they should provide the public with a clear action plan for the future, highlighting the future anxiety and fatigue that the Covid-19 pandemic is causing for everyone, trying to generate a sense of community and connectedness, and bolstering national attachment. This can be achieved only if all governmental representatives offer a coherent, clear, and unified interpretation of the facts and projections for the future.

## Data Availability

The data and materials that support the findings of this study are available from the corresponding author upon reasonable request.
